# Synthesis and Antimicrobial Activity of *N*′-Heteroarylidene-1-adamantylcarbohydrazides and (±)-2-(1-Adamantyl)-4-acetyl-5-[5-(4-substituted phenyl-3-isoxazolyl)]-1,3,4-oxadiazolines

**DOI:** 10.3390/molecules17033475

**Published:** 2012-03-16

**Authors:** Ali A. El-Emam, Khalid A. Alrashood, Mohamed A. Al-Omar, Abdul-Malek S. Al-Tamimi

**Affiliations:** Department of Pharmaceutical Chemistry, College of Pharmacy, King Saud University, Riyadh 11451, Saudi Arabia

**Keywords:** adamantane derivatives, 1,3,4-oxadiazoles, isoxazoles, antimicrobial activity

## Abstract

The reaction of adamantane-1-carbohydrazide (**1**) with heterocyclic aldehydes, namely 5-(4-chlorophenyl)isoxazole-3-carboxaldehyde (**2a**), 5-(4-methylphenyl)isoxazole-3-carboxaldehyde (**2b**), 5-(4-methoxyphenyl)isoxazole-3-carboxaldehyde (**2c**), 1*H*-imidazole-2-carboxaldehyde and 2-butyl-4-chloro-1*H*-imidazole-5-carboxaldehyde, in ethanol, yielded the corresponding *N*′-heteroarylidene-1-adamantylcarbohydrazides **3a**, **3b**, **3c**, **4** and **5**, respectively, in good yields. The 4-acetyl-1,3,4-oxadiazoline analogues **6a‑c** were prepared in 48–55% yields by heating their corresponding *N*′-heteroarylidene-1-adamantylcarbohydrazides **3a**–**c** with acetic anhydride for two hours. Compounds **3a**–**c**, **4**, **5** and **6a**–**c** were tested for *in vitro* activities against a panel of Gram-positive and Gram-negative bacteria and the yeast-like pathogenic fungus *Candida albicans*. Compounds **4** and **5** displayed potent broad-spectrum antimicrobial activity, while compounds **3a**–**c** showed good activity against the Gram-positive bacteria.

## 1. Introduction

The incorporation of an adamantyl moiety into a variety of molecules results in compounds with relatively high lipophilicity, which in turn can modify the biological availability of these molecules. In almost all cases, an adamantyl-bearing compound will be more lipophilic than the corresponding des-adamantyl analogue. Beyond increasing partition coefficients, the adamantyl group positively modulates the therapeutic index of many experimental compounds, through a variety of mechanisms [1,2]. Derivatives of adamantane have long been known for their antiviral activity against the influenza A [3,4,5,6] and HIV viruses [7,8,9]. Several adamantane derivatives were also associated with central nervous [10,11] and antimicrobial [12,13,14,15,16] properties. In addition, several 1,3,4-oxadiazole derivatives [17,18] and isoxazole derivatives [19,20] were reported to possess significant antimicrobial activity. In continuation to our interest in the chemical and pharmacological properties of adamantane derivatives [8,14,15,16,21,22,23,16,21], we report herein the synthesis, antimicrobial activity of new series of *N*’-heteroarylidene-1-adamantylcarbohydrazides and (±)-2-(1-adamantyl)-4-acetyl-5-[5-(4-substituted phenyl- 3-isoxazolyl)]-1,3,4-oxadiazolines.

## 2. Results and Discussion

### 2.1. Chemistry

**Scheme 1:**
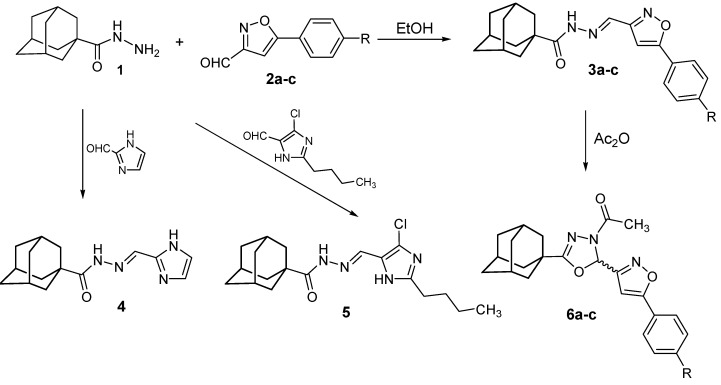
Synthesis of compounds **3a**–**c**, **4**, **5** and **6a**–**c**.

The adamantane-1-carbohydrazide (**1**) required as starting material, was prepared starting with adamantane-1-carboxylic acid *via* esterification with methanol to yield the methyl ester, which was subsequently reacted with hydrazine to yield adamantane-1-carboxylic acid hydrazide [21,24]. Adamantane-1-carbohydrazide (**1**) was next allowed to react in ethanol with heterocyclic aldehydes, namely 5-(4-chlorophenyl)isoxazole-3-carboxaldehyde (**2a**), 5-(4-methylphenyl)isoxazole-3-carbox-aldehyde (**2b**), 5-(4-methoxyphenyl)isoxazole-3-carboxaldehyde (**2c**), 1*H*-imidazole-2-carboxaldehyde and 2-butyl-4-chloro-1*H*-imidazole-5-carboxaldehyde, to yield the corresponding *N*′-heteroarylidene-1-adamantylcarbohydrazides **3a**–**c**, **4** and **5**, respectively, in good yield. The reaction of *N*-arylidene carboxylic acid hydrazides with acetic anhydride was reported to afford the corresponding *N*-acetyl-1,3,4-oxadiazoline derivatives [25,26,27,28]. Thus, compounds **3a**–**c** were successfully cyclized to their 4-acetyl-1,3,4-oxadiazoline analogues **6a**–**c** in 48–55% yield by heating with acetic anhydride for two hours (Scheme 1). 

Attempted reaction of the imidazole derivatives **4** or **5** with acetic anhydride under the same conditions failed to yield their *N*-acetyl-1,3,4-oxadiazoline derivatives. Meanwhile, increasing the reaction time up to four hours was also unsuccessful and the reactions afforded unidentified dark tarry materials. The structures of newly synthesized compounds **3a**–**c**, **4**, **5** and **6a**–**c** were confirmed by ^1^H-NMR, ^13^C-NMR and ESI-MS spectral data, in addition to the X-ray crystallography of compound **5** [29].

### 2.2. Antimicrobial Testing

The newly synthesized compounds **3a**–**c**, **4**, **5** and **6a**–**c** were tested for their* in vitro* growth inhibitory activity against the standard strains of the Institute of Fermentation of Osaka (IFO) namely; *Staphylococcus aureus* IFO 3060, *Bacillus subtilis* IFO 3007, *Micrococcus luteus* IFO 3232 (Gram-positive bacteria), *Escherichia coli* IFO 3301, *Pseudomonas aeuroginosa* IFO 3448 (Gram-negative bacteria), and the yeast-like pathogenic fungus *Candida albicans* IFO 0583. The primary screening was carried out using the agar disc-diffusion method using Müller-Hinton agar medium [30]. The results of the preliminary antimicrobial testing of compounds **3a**–**c**, **4**, **5** and **6a-c** (200 µg/8 mm disc), the antibacterial antibiotics gentamicin (100 µg/8 mm disc), ampicillin (100 µg/8 mm disc) and the antifungal drug clotrimazole (100 µg/8 mm disc) and the calculated log *P* values (Clog *P*) of the tested compounds (calculated using the CS ChemOffice Ultra, version 8.0, CambridgeSoft, Cambridge, MA, USA) are shown in Table 1.

**Table 1:** Antimicrobial activity of compounds **3a**–**c**, **4**, **5** and **6a**–**c** (200 µg/8 mm disc), the broad spectrum antibacterial antibiotics gentamicin (100 µg/8 mm disc), ampicillin (100 µg/8 mm disc) and the antifungal drug clotrimazole (100 µg/8 mm disc) against *Staphylococcus aureus *IFO 3060 (*SA*), *Bacillus subtilis *IFO 3007 (*BS*), *Micrococcus luteus* IFO 3232 (*ML*), *Escherichia coli *IFO 3301(*EC*), *Pseudomonas aeuroginosa *IFO 3448 (*PA*), and* Candida albicans *IFO 0583 (*CA*).

Comp. No.	Clog *P*	Diameter of Growth Inhibition Zone (mm) *					
		*SA*	*BS*	*ML*	*EC*	*PA*	*C A*
**3a**	4.96	19(16) ^†^	22(8) ^†^	17	11	-	-
**3b**	4.74	18(16) ^†^	18(16) ^†^	14	-	-	-
**3c**	4.24	17	18(16) ^†^	13	-	-	-
**4**	2.30	32(0.5) ^†^	26(0.5) ^†^	23(8) ^†^	16	11	17
**5**	3.89	28(1) ^†^	30(1) ^†^	22(4) ^†^	19(8) ^†^	14	16
**6a**	5.20	12	11	11	-	-	-
**6b**	4.95	-	-	-	-	-	-
**6c**	4.48	-	-	-	-	-	-
**Gentamicin**		26(2) ^†^	25(2) ^†^	18(2) ^†^	20(0.5) ^†^	19(1) ^†^	NT
**Ampicillin**		23(2) ^†^	21(0.5) ^†^	19(2) ^†^	17(2) ^†^	16(2) ^†^	NT
**Clotrimazole**		NT	NT	NT	NT	NT	21

* (-): Inactive (inhibition zone ≤ 10 mm); (NT): Not tested; † The figures shown in parentheses represent the MIC values (µg/mL).

The antimicrobial activity results of the synthesized compounds (Table 1) revealed that the tested compounds showed varying degrees of inhibition against the tested microorganisms. In general, it was observed that the antibacterial activity is not dependent on their lipophilicity. The antibacterial activity of the tested *N*’-heteroarylidene-1-adamantylcarbohydrazides **3a**–**c**, **4** and **5 **mainly depended on the heterocyclic nucleus. The imidazole derivatives **4** and **5** displayed higher and broad-spectrum activity than the isoxazole derivatives **3a**–**c**, which exhibited potent or moderate activity against the tested Gram-positive bacteria. The cyclization of the *N*’-heteroarylidene analogues **3a**–**c** to their 4-acetyl-1,3,4-oxadiazoline analogues **6a**–**c** resulted in a dramatic decrease in the antibacterial activity, and only compound **6a** retained marginal activity against the tested Gram-positive bacteria (Table 1). The minimal inhibitory concentration (MIC) for the active compounds **3a**–**c**, **4** and **5** against the same microorganism used in the primary screening was carried out using the microdilution susceptibility method in Müller-Hinton Broth and Sabouraud Liquid Medium [31]. The MIC of the compounds **3a**-**c**, **4** and **5**, the antibacterial antibiotics gentamicin and ampicillin trihydrate which are shown in Table 1, were in accordance with the results obtained in the primary screening.

## 3. Experimental

### 3.1. General

Melting points (°C) were measured in open glass capillaries using a Branstead 9001 electrothermal melting point apparatus and are uncorrected. NMR spectra were obtained on a Brüker AC 500 Ultra Shield NMR spectrometer (Brüker BioSpin AG, Fällanden, Switzerland) operating at 500.13 MHz for ^1^H and 125.76 MHz for ^13^C; the chemical shifts are expressed in δ (ppm) downfield from tetramethylsilane (TMS) as internal standard; coupling constants (*J*) are expressed in Hz. Electrospray ionization mass spectra (ESI-MS) were recorded on an Agilent 6410 Triple Quad tandem mass spectrometer (Agilent Technologies, Palo Alto, CA, USA) at 4.0 and 3.5 kV for positive and negative ions, respectively. Elemental analyses (C, H, N) were in full agreement with the proposed structures within ±0.4% of the theoretical values. Monitoring the reactions and checking the purity of the final products were carried out by thin layer chromatography (TLC) using silica gel precoated aluminum sheets (60 F_254_, Merck) and visualization with ultraviolet light (UV) at 365 and 254 nm. The bacterial strains and *Candida albicans* fungus were obtained from the Institute of Fermentation of Osaka (IFO, Osaka, Japan). The reference drugs gentamicin (CAS 1405-41-0), ampicillin trihydrate (CAS 7177-48-2) and clotrimazole (CAS 23593-75-1) were obtained from Sigma-Aldrich Chemie GmbH (Taufkirchen, Germany).

### 3.2. N′-Heteroarylidene-1-adamantylcarbohydrazides **3a–c**, **4** and **5**

The appropriate heterocyclic aldehyde **2a**–**c** (0.01 mol) was added to a solution of adamantane-1-carbohydrazide **1** (1.94 gm, 0.01 mol) in ethanol (10 mL), and the mixture was heated under reflux for 3 hours and allowed to stand at room temperature for overnight. The separated precipitate was filtered, washed with water, dried and crystallized from ethanol (compounds **3a**–**c**) or aqueous ethanol (compounds **4**, **5**).

N′-*[5-(4-Chlorophenyl)isoxazol-3-yl)methylidene]adamantane-1-carbohydrazide * (**3a**): M.p.: 223–225 °C, Yield: 3.46 gm (90%). ^1^H-NMR (DMSO-d_6_): δ 1.71 (s, 6H, adamantane-H), 1.89 (s, 6H, adamantane-H), 2.02 (s, 3H, adamantane-H), 7.37 (s, 1H, isoxazole-H), 7.60 (d, 2H, Ar-H, *J* = 8.0 Hz), 7.98 (d, 2H, Ar-H, *J* = 8.0 Hz), 8.52 (s, 1H, CH=N), 11.21 (s, 1H, NH). ^13^C-NMR: δ 27.48, 35.95, 38.13, 39.99 (adamantane-C), 97.82, 161.25, 168.36 (isoxazole-C), 125.28, 127.58, 129.31, 135.29 (Ar-C), 136.14 (CH=N), 173.52 (C=O). ESI-MS, *m/z* (Rel. Int.): 384.9 (M^−^ +2, 34), 382.9 (M^−^, 100).

N′-*[5-(4-Methylphenyl)isoxazol-3-yl)methylidene]adamantane-1-carbohydrazide * (**3b**): M.p.: 201–203 °C, Yield: 3.13 gm (86%). ^1^H-NMR (DMSO-d_6_): δ 1.71 (s, 6H, adamantane-H), 1.89 (s, 6H, adamantane-H), 2.02 (s, 3H, adamantane-H), 2.37 (s, 3H, CH_3_), 7.22 (s, 1H, isoxazole-H), 7.34 (d, 2H, Ar-H, *J* = 7.5 Hz), 7.83 (d, 2H, Ar-H, *J* = 7.5 Hz), 8.52 (s, 1H, CH=N), 11.20 (s, 1H, NH). ^13^C-NMR: δ 20.97 (CH_3_), 27.18, 35.95, 38.14, 38.98 (adamantane-C), 96.51, 161.08, 169.64 (isoxazole-C), 123.78, 125.70, 129.74, 136.36 (Ar-C), 140.55 (CH=N), 173.53 (C=O). ESI-MS, *m/z* (Rel. Int.): 362.2 (M^−^, 100).

N′-*[5-(4-Methoxyphenyl)isoxazol-3-yl)methylidene]adamantane-1-carbohydrazide * (**3c**): M.p.: 199–201 °C, Yield: 3.26 gm (86%). ^1^H-NMR (DMSO-d_6_): δ 1.71 (s, 6H, adamantane-H), 1.89 (s, 6H, adamantane-H), 2.02 (s, 3H, adamantane-H), 3.83 (s, 3H, OCH_3_), 7.08 (d, 2H, Ar-H, *J* = 7.0 Hz), 7.15 (s, 1H, isoxazole-H), 7.89 (d, 2H, Ar-H, *J* = 7.0 Hz), 8.51 (s, 1H, CH=N), 11.19 (s, 1H, NH). ^13^C-NMR: δ 27.48, 35.95, 38.14, 38.98 (adamantane-C), 55.34 (OCH_3_), 95.60, 161.05, 169.54 (isoxazole-C), 114.61, 119.12, 127.49, 160.98 (Ar-C), 136.44 (CH=N), 173.51 (C=O). ESI-MS, *m/z* (Rel. Int.): 378.2 (M^−^, 100).

N′-*[(1H-Imidazol-2-yl)methylidene]adamantane-1-carbohydrazide * (**4**): M.p.: 210–212 °C, Yield: 1.69 gm (62%). ^1^H-NMR (DMSO-d_6_): δ 1.70–1.76 (m, 6H, adamantane-H), 1.88–1.91 (m, 6H, adamantane-H), 2.01 (s, 3H, adamantane-H), 7.08–7.42 (m, 2H, imidazole-H), 8.31 (s, 1H, CH=N), 10.81 (s, 1H, NH), 13.67 (s, 1H, imidazole-NH). ^13^C-NMR: δ 27.53, 35.99, 38.29, 38.68 (adamantane-C), 128.95, 129.37, 138.58 (imidazole-C), 141.33 (CH=N), 173.10 (C=O). ESI-MS, *m/z* (Rel. Int.): 271.1 (M^−^, 100).

N′*-[(2-Butyl-4-chloro-1H-imidazol-5-yl)methylidene]adamantane-1-carbohydrazide * (**5**): M.p.: 188.90 °C, Yield: 2.58 gm (71%). ^1^H-NMR (DMSO-d_6_): δ 0.86 (t, 3H, CH_3_, *J* = 7.0 Hz), 1.05–1.08 (m, 2H, C***H***_2_CH_3_), 1.25–1.29 (m, 2H, C***H***_2_CH_2_CH_3_), 1.58–1.61 (m, 2H, C***H***_2_CH_2_CH_2_CH_3_), 1.69 (s, 6H, adamantane-H), 1.86 (s, 6H, adamantane-H), 2.0 (s, 3H, adamantane-H), 8.27 (s, 1H, CH=N), 10.72 (s, 1H, NH), 12.76 (s, 1H, imidazole-NH). ^13^C-NMR: δ 13.51 (CH_3_), 18.49 (***C***H_2_CH_3_), 21.51 (***C***H_2_CH_2_CH_3_), 27.28 (***C***H_2_CH_2_CH_2_CH_3_), 27.56, 29.80, 36.01, 38.33 (adamantane-C), 120.73, 130.45, 134.97 (imidazole-C), 150.67 (CH=N), 172.91 (C=O). ESI-MS, *m/z* (Rel. Int.): 363.2 (M^−^ +2, 30), 361.2 (M^−^, 100).

### 3.3. (±)-2-(1-Adamantyl)-4-acetyl-5-[5-(4-substituted phenyl-3-isoxazolyl)]-1,3,4-oxadiazolines **6a–c**

A mixture of the appropriate *N’*-heteroarylidene-1-adamantylcarbohydrazides **3a**–**c** (5.0 mmol) and acetic anhydride (8 mL) was heated under reflux for 2 hours. The excess acetic anhydride was then evaporated under reduced pressure and ice-water (50 mL) was added to the resulted oily or sticky residue and refrigerated for 2 hours. The separated solid was filtered, washed with water, dried and crystallized from aqueous ethanol to yield the products **6a**–**c** in 48–55% yield.

*(±)-2-(1-Adamantyl)-4-acetyl-5-[5-(4-chlorophenyl-3-isoxazolyl)]-1,3,4-oxadiazoline* (**6a**): M.p.: 131–133 °C, Yield: 2.34 gm (55%). ^1^H-NMR (DMSO-d_6_): δ 1.70 (br. s, 3H, adamantane-H), 1.89–2.01 (m, 12H, adamantane-H), 2.09 (s, 3H, COCH_3_), 7.18 (s, 1H, isoxazole-H), 7.19 (s, 1H, oxadiazole-H), 7.62 (d, 2H, Ar-H, *J* = 8.5 Hz), 7.95 (d, 2H, Ar-H, *J* = 8.5 Hz). ^13^C-NMR: δ 21.02 (CO***C***H_3_), 26.93, 30.66, 35.67, 38.38 (adamantane-C), 83.96, 163.50 (oxadiazole-C), 98.62, 164.50, 169.50 (isoxazole-C), 124.50, 127.57, 120.37, 134.50 (Ar-C), 171.98 (C=O). ESI-MS, *m/z* (Rel. Int.): 451.3 (M^+^ +2+Na, 22), 449.3 (M^+^ +Na, 100).

*(±)-2-(1-Adamantyl)-4-acetyl-5-[5-(4-methylphenyl-3-isoxazolyl)]-1,3,4-oxadiazoline* (**6b**): M.p.: 126–128 °C, Yield: 2.07 gm (51%). ^1^H-NMR (DMSO-d_6_): δ 1.69 (s, 6H, adamantane-H), 1.86 (s, 6H, adamantane-H), 1.99 (s, 3H, adamantane-H), 2.17 (s, 3H, COCH_3_), 2.37 (s, 3H, CH_3_), 7.03 (s, 1H, isoxazole-H), 7.16 (s, 1H, oxadiazole-H), 7.34 (d, 2H, Ar-H, *J* = 7.5 Hz), 7.79 (d, 2H, Ar-H, *J* = 7.5 Hz). ^13^C-NMR: 20.91 (CO***C***H_3_), 20.97 (CH_3_), 26.94, 33.44, 35.69, 38.37 (adamantane-C), 83.68, 161.06 (oxadiazole-C), 97.33, 163.87, 168.59 (isoxazole-C), 123.64, 125.66, 129.76, 140.72 (Ar-C), 170.52 (C=O). ESI-MS, *m/z* (Rel. Int.): 429.3 (M^+^ +1+Na, 100), 406.3 (M^+^, 22).

*(±)-2-(1-Adamantyl)-4-acetyl-5-[5-(4-methoxyphenyl-3-isoxazolyl)]-1,3,4-oxadiazoline* (**6c**): M.p.: 119–121 °C, Yield: 2.02 gm (48%). ^1^H-NMR (DMSO-d_6_): δ 1.70 (s, 6H, adamantane-H), 1.89 (s, 6H, adamantane-H), 2.0–2.02 (m, 3H, adamantane-H), 2.17 (s, 3H, COCH_3_), 3.83 (s, 3H, OCH_3_), 6.95 (s, 1H, isoxazole-H), 7.08 (s, 1H, oxadiazole-H), 7.14 (d, 2H, Ar-H, *J* = 8.5 Hz), 7.89 (d, 2H, Ar-H, *J* = 8.5 Hz). ^13^C-NMR: 20.0 (CO***C***H_3_), 27.48, 33.44, 35.95, 38.14 (adamantane-C), 55.36 (OCH_3_), 83.71, 163.89 (oxadiazole-C), 96.39, 166.49, 169.55 (isoxazole-C), 114.62, 118.97, 127.49, 161.06 (Ar-C), 173.53 (C=O). ESI-MS, *m/z* (Rel. Int.): 422.4 (M^+^, 100).

### 3.4. Determination of the Antimicrobial Activity by the Agar Disc-Diffusion Method [30]

Sterile filter paper discs (8 mm diameter) were moistened with the compound solution in dimethylsulphoxide of specific concentration (200 µg/disc), the antibacterial antibiotics gentamicin and ampicillin trihydrate (100 µg/disc) and the antifungal drug clotrimazole (100 µg/disc) were carefully placed on the agar culture plates that had been previously inoculated separately with the microorganisms. The plates were incubated at 37 °C, and the diameter of the growth inhibition zones were measured after 24 hours in case of bacteria and 48 h in case of *Candida albicans*.

### 3.5. Determination of Minimal Inhibitory Concentrations (MICs) [31]

Compounds **3a**–**c**, **4**, **5**, gentamicin and ampicillin trihydrate were dissolved in dimethylsulphoxide at the concentration of 128 µg/mL. The twofold dilutions of the solution were prepared (128, 64, 32, 16, 8, 4, 2, 1 and 0.5 µg/mL). The microorganism suspensions at 106 CFU/mL (colony forming unit/mL) concentrations were inoculated to the corresponding wells. The plates were incubated at 36 °C for 24. The MIC values were determined as the lowest concentration that completely inhibited visible growth of the microorganism as detected by unaided eye.

## 4. Conclusions

In this study, the synthesis and characterization of a series of *N*′-heteroarylidene-1-adamantylcarbohydrazides (compounds **3a**–**c**, **4**, **5**) and 2-(1-adamantyl)-4-acetyl-5-[5-(4-substituted phenyl-3-isoxazolyl)]-1,3,4-oxadiazolines (compounds **6a**–**c**) was described. The structure-antimicrobial activity relationship studies revealed that the *N*’-heteroarylidene-1-adamantyl-carbohydrazides were viable leads for further studies.
